# Diversity of the gut microbiota and eczema in early life

**DOI:** 10.1186/1476-7961-6-11

**Published:** 2008-09-22

**Authors:** Erick Forno, Andrew B Onderdonk, John McCracken, Augusto A Litonjua, Daniel Laskey, Mary L Delaney, Andrea M DuBois, Diane R Gold, Louise M Ryan, Scott T Weiss, Juan C Celedón

**Affiliations:** 1 Channing Laboratory Boston, MA, USA; 2 Division of Pulmonary/Critical Care Medicine, Dept. of Medicine, Brigham and Women's Hospital, Boston, MA, USA; 3 Harvard Medical School, Boston, MA, USA; 4 Division of Pediatric Pulmonology, Dept. of Pediatrics, Children's Hospital, Boston, MA, USA; 5 Dept. of Pathology, Brigham and Women's Hospital, Boston, MA, USA; 6 Dept. of Biostatistics, Harvard School of Public Health, Boston, MA, USA

## Abstract

**Background:**

A modest number of prospective studies of the composition of the intestinal microbiota and eczema in early life have yielded conflicting results.

**Objective:**

To examine the relationship between the bacterial diversity of the gut and the development of eczema in early life by methods other than stool culture.

**Methods:**

Fecal samples were collected from 21 infants at 1 and 4 months of life. Nine infants were diagnosed with eczema by the age of 6 months (cases) and 12 infants were not (controls). After conducting denaturating gradient gel electrophoresis (DGGE) of stool samples, we compared the microbial diversity of cases and controls using the number of electrophoretic bands and the Shannon index of diversity (*H'*) as indicators.

**Results:**

Control subjects had significantly greater fecal microbial diversity than children with eczema at ages 1 (mean *H' *for controls = 0.75 vs. 0.53 for cases, P = 0.01) and 4 months (mean *H' *for controls = 0.92 vs. 0.59 for cases, P = 0.02). The increase in diversity from 1 to 4 months of age was significant in controls (P = 0.04) but not in children who developed eczema by 6 months of age (P = 0.32).

**Conclusion:**

Our findings suggest that reduced microbial diversity is associated with the development of eczema in early life.

## Background

Eczema is often the first manifestation of atopy in infants who will develop asthma or allergic rhinitis later in childhood [1]. The prevalence of atopic diseases such as eczema has been on the rise for several decades, particularly in industrialized nations [2,3]. Potential explanations for the increased prevalence of eczema and other atopic diseases include reduced exposure to microbial agents (the "hygiene hypothesis"[4]) and/or changes in the gut microbiota in early life [5].

Few prospective studies have examined the relation between the composition of the gut microbiota in early life and atopy [6-9]. A study of 324 European infants followed from birth to age 18 months found that neither time to gut colonization with 11 bacterial groups nor ratio of strict anaerobic to facultative anaerobic bacteria in cultures from neonatal stool samples was associated with eczema or food allergy [7]. In contrast, a study of 957 Dutch infants showed that the presence of *C. difficile *in stool samples at age 1 month (assessed by quantitative real-time PCR) was associated with increased risks of eczema, recurrent wheeze, and allergic sensitization at age 2 years [8]. In that study, early colonization with *E. coli *was associated with eczema by parental report but not with objectively diagnosed eczema. The conflicting results of these studies may be due to differences in statistical power and/or limited ability to adequately culture the complex gut microbiota [10].

Novel methods such as analysis of bacterial 16S ribosomal DNA [rDNA] (using universal primers or denaturating techniques) [11,12] are much more sensitive for detecting certain bacterial species in the mouth or gut than traditional cultures. Using temporal temperature gradient gel electrophoresis (TTGE), Li and colleagues found a significant difference in the diversity of the oral microbiota of children with and without severe dental caries [13]. Using a similar approach, Wang and colleagues recently reported that reduced diversity of the gut microbiota at age 1 week is associated with eczema in the first 18 months of life [9].

We performed denatured gradient gel electrophoresis (DGGE) analysis of bacterial 16S rDNA genotypes on stool samples to assess whether the microbial diversity of the gut microbiota at ages 1 and 4 months is associated with the development of eczema in early life. We report that reduced microbial diversity of the neonatal gut microbiota in the first 4 months of life is associated with an increased risk of eczema at age 6 months.

## Methods

### Study cohort

Pregnant women were recruited between July 2003 and November 2005 from three outpatient facilities affiliated with Brigham and Women's Hospital in Boston at their 24-week prenatal visit, as previously described [14]. Inclusion criteria were maternal age between 18 years and 44 years; plans to deliver at Brigham and Women's Hospital; and maternal ability to speak English or Spanish. Informed consent was obtained from participating mothers. Of the 37 participating neonates, nine had been diagnosed with eczema by a physician before age 6 months and were included as cases for this analysis. Twelve healthy children (no diagnosis of eczema) were then matched to the cases on gender and included as control subjects for this study. The study was approved by the Institutional Review Board of Brigham and Women's Hospital.

A questionnaire was administered to each participating woman between her 24-week prenatal visit and delivery to obtain information on demographics, general health, and history of allergic diseases and/or symptoms for each of the child's parents.

Information on labor and delivery was obtained from review of medical records. When the child was 2 and 6 months of age, a telephone questionnaire (modified from the American Thoracic Society-Division of Lung Diseases questionnaire[15]) was administered by trained research assistants to the child's primary caretaker.

### Stool collection and denatured gradient gel electrophoresis (DGGE) analysis of bacterial 16S rDNA in stool samples

A stool sample was collected from participating neonates at ages 1 and 4 months. More than a gram of stool was collected into a sterile specimen container and frozen for transport to the laboratory. Approximately 0.05 gram of stool specimen was placed into a 1.5 ml sterile tube. Following the fecal DNA purification protocol supplied by the manufacturer, DNA was extracted using the ExtractMaster Fecal DNA extraction kit (EPICENTRE Biotechnologics, Madison, WI). The V2–V3 region of the 16S rDNA gene of bacteria in the fecal samples was amplified using the primers described by Tannock et al [16]. PCR was performed using a Biorad thermal cycler and 0.2 ml tubes. The reaction mixture (50 μL) contains Platinum PCR SuperMix High Fidelity (Invitrogen, Carlsbad CA), 1 unit of Platinum Taq DNA Polymerase High Fidelity (Invitrogen), 20 pmol of each primer and 4 μl of fecal DNA. Amplification was 94°C for 3 minutes, 30 cycles of 94°C for 30 s, 56°C for 30 s, 68°C for 60 s and 7 minutes at 68°C at the end of the cycles. DGGE was performed using the DCode universal mutation detection system (Bio-Rad Laboratories, Hercules CA) in gels that were 16 cm × 16 cm by 1 mm; 6% polyaccrylamide gels were prepared and electrophoresed with 1× tris-acetate EDTA (TAE) buffer prepared from 50× TAE buffer. The denaturing gradient was formed by using two 6% polyacrylamide stock solutions containing a 20–55% urea/formamide gradient that increases in the direction of electrophoresis. A 100% denaturing solution containing 40% formamide and 7.0 M urea was used. Electrophoresis was performed at 130 V and 60°C for approximately 4 hours. The electrophoresis was stopped when a xylene cyanol dye marker reached the bottom of the gel. Gels were stained with ethidium bromide solution (5 μg/mL) for 20 minutes, washed with deionized water and viewed using a Gel Doc UV transillumination system (Bio-Rad).

### Statistical analysis

The number of DGGE bands was used as an indicator of fecal bacterial diversity for each participating child, both at 1 and at 4 months of age. Densitometric curves were obtained for all the bands in each sample, and the relative intensity of each band in the sample (*p*_*i*_) was computed. Shannon diversity indices (*H'*) were then calculated for each patient at 1 and 4 months of age, using the formula *H' *= -Σ *p*_i_* ln(*p*_*i*_)[17].

Univariate analysis for baseline characteristics was performed using Mann-Whitney rank-sum tests. The differences in bacterial diversity between cases and controls at each point in time (1 and 4 months of age) were tested by Mann-Whitney rank-sum tests and by logistic regression modeling; non-parametric analysis was repeated after stratification by mode of delivery. We explored the behavior of bacterial diversity over time using mixed effects linear modeling in order to account for correlations between repeated measures on the same subjects [18]. An interaction term between time and eczema status was included to assess the differential effect of time in the two groups. All statistical analysis was performed using SAS v9.1 (SAS Institute Inc., Cary, NC).

## Results

Table 1 shows the main characteristics of participating children. Approximately half of the children were girls (by matching design) who were born by vaginal delivery and who were breastfed. There were no significant differences in breastfeeding, mode of delivery, birthweight, 5-minute APGAR score, day care attendance, or use of antibiotics between children who did and did not develop eczema by age 6 months.

**Table 1:** Characteristics of participating children

	Total n (%)	Eczema^1 ^n (%)	Controls n (%)	P-value^2^
Gender:				
-Male	11 (52.4)	4 (44.4)	7 (58.3)	0.67
Mode of delivery:				0.67
-Vaginal	10 (47.6)	5 (55.6)	5 (41.7)	
-Cesarean	11 (52.4)	4 (44.4)	7 (58.3)	
Maternal history of atopy	12 (57.1)	7 (77.8)	5 (41.7)	0.18
Term delivery^3^	17 (81.0)	7 (77.8)	10 (83.3)	1.00
Birthweight (g), mean (SD)	3319 (688)	3192 (395)	3415 (851)	0.24
5-minute APGAR, median	9	9	9	0.50
Breastfed	11 (52.4)	6 (66.7)	5 (41.7)	0.39
Daycare attendance	5 (23.8)	1 (11.1)	4 (33.3)	0.34
Use of antibiotics^4^	3 (18.8)	1 (16.7)	2 (20.0)	1.00
**Measures of bacterial diversity:**				

**DGGE bands**^5^, mean (95%CI):				
-1 month of age	4.4 (3.6–5.1)	3.8 (2.6–4.9)	4.8 (3.8–5.9)	0.10
-4 months of age	5.3 (3.8–6.7)	3.9 (2.3–5.4)	6.5 (4.2–8.8)	**0.06**
**Shannon index**^6^, mean (95%CI):				
-1 month of age	0.66 (0.55–0.76)	0.53 (0.38–0.67)	0.75 (0.63–0.88)	**0.01**
-4 months of age	0.77 (0.62–0.91)	0.59 (0.38–0.81)	0.92 (0.76–1.08)	**0.02**

^1 ^Defined as parental report of physician-diagnosed eczema at or before 6 months of age. ^2^Mann-Whitney P-values for the comparison between cases with eczema and controls.

^3^Birth between 37 and 41 weeks of gestation.

^4^Maternal report of antibiotic administration to the child

^5^Number of denaturating gradient gel electrophoresis (DGGE) bands in each sample

^6^Shannon *H' *index calculated using the relative intensity of all bands in each sample

**Figure 1:**
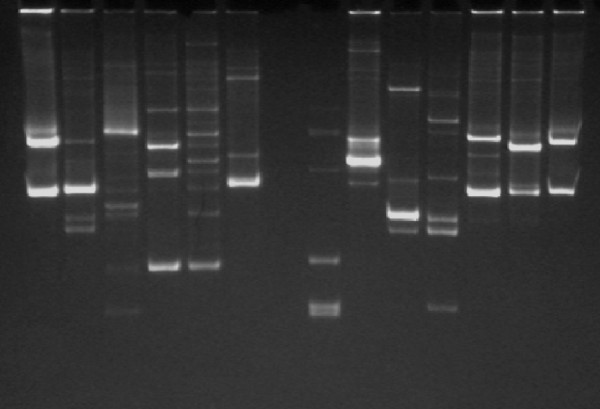
**Denaturating gradient gel electrophoresis (DGGE)**. Stool samples were processed to extract 16S rDNA, the V2–V3 region was amplified by PCR, and denaturating gradient gel electrophoresis (DGGE) was performed using a standard protocol. For each lane, representing a single sample, the number of bands was counted and the Shannon index of diversity *H' *was calculated.

**Figure 2:**
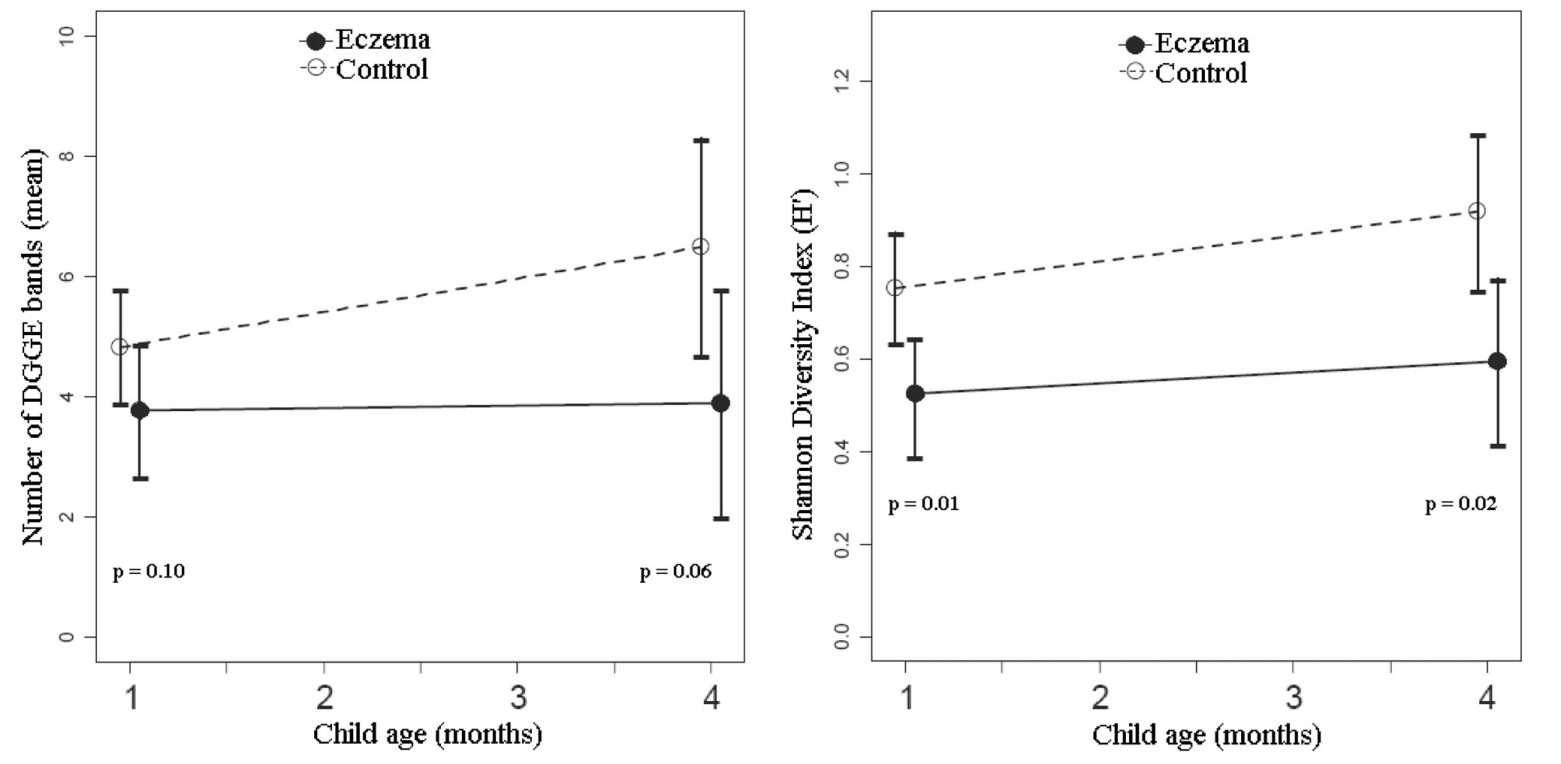
**Number of bands and Shannon diversity index in all subjects**. Controls had a higher number of bands at age 4 months (p = 0.06), and a higher Shannon index both at 1 (p = 0.01) and 4 months of age (p = 0.02).

The results of the analysis of the mean number of DGGE bands (representing bacterial 16S rDNA profiles, see Figure 1) and the Shannon diversity indices in participating children are included in Table 1 and illustrated in Figure 2. There was a trend for a higher mean number of bands in controls than in children with eczema at 1 month of life, with a more pronounced difference at 4 months. Similarly, controls had significantly higher indices of diversity (*H'*) than cases, both at 1 and at 4 months. The observed differences in the index of diversity at ages 1 and 4 months remained statistically significant after adjustment for mode of delivery and maternal history of atopy (data not shown).

We then examined the relation between increasing bacterial diversity and eczema in bivariate analyses using logistic regression (Table 2). At age 1 month, an increment in the Shannon diversity index from the mean value for cases (0.53) to the mean value for controls (0.75) was associated with a 70% reduction in the odds of eczema by age 6 months. There was a similar yet nonstatistically significant trend for an inverse association between the number of electrophoretic bands and eczema. At age 4 months, an increment in the number of bands from the mean value for cases (3.9) to the mean value for controls (6.5) was associated with a 75% reduction in the odds of eczema by age 6 months. In addition, an increment in the Shannon diversity from the mean value for cases (0.59) to the mean value for controls (0.92) at age 4 months was associated with an 85% reduction in the odds of eczema. The results of analyses of microbial diversity and eczema were not appreciably changed after adjustment for relevant covariates (Table 2).

**Table 2:** Fecal microbial diversity at ages 1 and 4 months, and eczema^1 ^at age 6 months.

		Odds ratio^2 ^(95% CI)	
		
	Age	Unadjusted	Adjusted^3^
**DGGE bands**^4^	1 month	0.61 (0.28–1.13)	0.52 (0.19–1.08)
	4 months	0.25 (0.04–0.87)	0.19 (0.01–0.87)
			
**Shannon index**^5^	1 month	0.30 (0.08–0.80)	0.23 (0.04–0.71)
	4 months	0.15 (0.01–0.66)	0.08 (0.01–0.58)

^1^Defined as maternal report of physician-diagnosed eczema at or before 6 months of age

^2^Odds ratio of having eczema if fecal microbial diversity (number of bands or Shannon index) increases from the mean value for cases to the mean value for controls (e.g. from 3.8 to 4.8 bands at 1 month, etc).

^3^Adjusted for mode of delivery and maternal history of atopy. Adjusted odds ratios were not significantly different than unadjusted ones.

^4^Number of denaturating gradient gel electrophoresis (DGGE) bands in each sample

^5^Shannon diversity index (*H'*) calculated using the relative intensity of all bands within each sample

**Figure 3:**
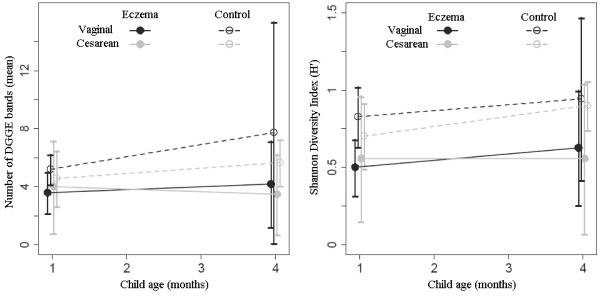
**Number of bands and Shannon diversity index by mode of delivery: **Controls delivered vaginally had a higher Shannon index than cases at 1 month (p = 0.04) but not at 4 months; index for controls delivered by C-section was similar to cases at 1 month, but higher at 4 months (p = 0.04).

Because of the known influence of mode of delivery on the neonatal gut microbiota, we repeated the analysis after stratification by mode of delivery (Table 3 and Figure 3). Among children delivered vaginally, both measures of diversity were significantly higher in controls than in cases at the age of 1 month, with a non-statistically significant difference at 4 months. Among children born by cesarean section, controls had a significantly higher Shannon index at age 4 months of age, with no significant difference at 1 month. Odds ratios were not calculated due to the small number of subjects in each subgroup.

**Table 3:** Fecal microbial diversity at ages 1 and 4 months, and eczema^1 ^at age 6 months, stratified by mode of delivery

		**Vaginal Delivery**^2^			**Cesarean Section**^2^		
		
		Means			Means		
					
	Age	Eczema	Controls	p-value^3^	Eczema	Controls	p-value^3^
**DGGE bands**^4^	1 month	3.60	5.20	0.05	4.00	4.57	0.75
	4 months	4.20	7.75	0.26	3.50	5.67	0.12

**Shannon index**^5^							
	1 month	0.50	0.83	0.04	0.56	0.70	0.16
	4 months	0.63	0.94	0.18	0.56	0.90	0.04

^1^Defined as parental report of physician-diagnosed eczema at or before 6 months of age

^2^There was no difference in microbial diversity when comparing delivery modes regardless of eczema status (p > 0.90)

^3^Mann-Whitney *U*-test p-value

^4^Number of denaturating gradient gel electrophoresis (DGGE) bands in each sample

^5^Shannon diversity index (*H'*) calculated using the relative intensity of all bands within each sample

**Figure 4:**
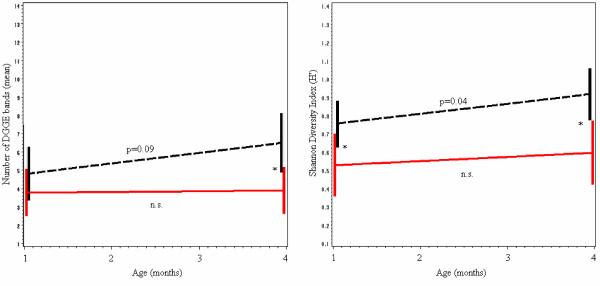
**Predictions from mixed effects linear regression models**. (Note: cases in red; controls in black). Number of bands: The increase in the number of bands tends to be more substantial in controls (p = 0.09) than in cases (p = 0.87); by age 4 months, controls have on average 2.6 bands more than cases. Shannon index: *H' *increases significantly in controls (p = 0.04) but not in cases (p = 0.32); at 1 month of age the index for controls is 0.22 higher, and at 4 months it is 0.33 higher. (*p < 0.05; see Table 1).

Mixed effects linear regression modeling was used to directly evaluate the behavior of bacterial diversity over time, with an interaction term included to assess whether the effect of time differed between cases and controls. Both models are illustrated in Figure 4. For the number of bands, there was no difference between cases and controls at 1 month of age. Controls acquired an average of 1.3 bands from age 1 month to age 4 months (p = 0.09), whereas cases only increased by 0.1 bands (p = 0.87); this resulted in a significant difference by age 4 months, with controls having on average 2.6 more bands than cases (p = 0.04). For the Shannon index model, controls had a significantly higher diversity than cases at 1 and 4 months of age, as previously described. During that period, *H' *increased an average of 0.11 among controls (p = 0.04), whereas the increase among cases was not significant (p = 0.32).

## Discussion

DGGE is a method to assess bacterial microbiota that is culture-independent and is based on electrophoresis of denaturated bacterial 16S rDNA genotypes. Using the number of electrophoretic bands and the Shannon index as markers of gut microbial diversity, we found that a reduced fecal bacterial diversity is associated with an increased risk of physician-diagnosed eczema in early life. These findings extend those of a recent report by Wang and colleagues of an inverse association between bacterial diversity of the gut microbiota at 1 week of age and eczema diagnosed by the age of 18 months in European infants, using terminal restriction fragment length polymorphism (T-RFLP) and temporal temperature gradient electrophoresis (TTGE)[9]. Our results suggest that differences in microbial diversity of the gut between children who will and will not develop eczema in infancy persist up to age 4 months and in fact increase during the first months of life.

An inverse association between family size and hay fever led to the hypothesis that reduced microbial exposure in early life increases the risk of developing allergic diseases (the "hygiene hypothesis") [4,19]. Several potential mechanisms have been proposed. T_H_2 cells promote allergen-specific responses via cytokines that increase production of IgE, eosinophilia, and mast cell proliferation [20,21]. In neonates, the immune system is skewed towards a T_H_2 profile [22], and it has been postulated that a decreased microbial exposure increases the risk of atopy because of an insufficient shift towards a more balanced T_H_1/T_H_2 response ("missing immune deviation")[20,23]. T_REG _cells, a newly characterized group of immune-modulatory T cells, suppress T_H_1 and T_H_2 activity by several mechanisms [24-26]. Early antigen exposure may influence T_REG _activity [27].

To date, no specific microbe or microbial product responsible for these observations has been confidently identified. The gut is the most important source of postnatal microbial stimulation of the immune system [28]. In experimental models, neonate mice with sterile gastrointestinal tracts do not develop a normal T_H_1/T_H_2 balance [29]; reintroduction of bacteria normalizes such balance [30,31]. Exposure of murine T_REG _cells to lipopolysaccharide stimulates their activity by expression of Toll-like receptors [32]. Murosaki [33] and Shida [34] have demonstrated that certain species of *Lactobacillus *decrease *in vivo *production of IgE in mice. Recently, Forsythe and colleagues reported that oral *L. reuteri *decreased eosinophilia and allergen-induced airway responsiveness in a murine model [35].

Human studies of the gut microbiota and allergies have yielded conflicting results. Adlerberth et al. found no association between any of 11 fecal bacterial groups and atopy in a cohort of European children [7]. On the other hand, Penders and colleagues found an association between *C. difficile *in stool samples at age 1 month and several markers of atopy in 957 Dutch infants [8]. Small clinical trials have found that oral supplementation of probiotics containing different species of lactobacilli and bifidobacteria result in reduced severity of eczema [36-38]. Both murine models and human studies have failed to isolate specific bacteria associated with atopy in a consistent manner. Rather than attempting to reduce the cause of these abnormalities in the immune system to the level of a specific causative organism, a systems biology approach would suggest the key factor is the alteration of the gut microbiota as a whole.

An alternate plausible explanation for the observed association between an anomalous gut microbiota and eczema is that the abnormalities in the immune system that lead to the disease (e.g., failure to develop a balanced T_H_1/T_H_2 response by age 1 year, lack of a normal T_REG _activity, etc.) prevent the acquisition or preservation of a "normally diverse" gut microbiota. The decreased bacterial diversity and the development of eczema would be parallel consequences of the same underlying mechanisms. However, a causal relationship is supported both by experimental data demonstrating the restitution of normal immune function and decreased inflammation in mice after reintroduction of bacteria [30-35], and by human studies demonstrating prevention or improvement of atopic dermatitis in infants after probiotic administration [36-39].

Of interest, a reduced microbial diversity has been implicated in various diseases. Li and colleagues found that the microbial diversity of the oral cavity was significantly reduced in children with severe early-childhood caries[40] when compared to controls. A potential explanation for this finding is that certain groups of bacteria supplant or dominate the plaque flora in individuals with caries, whereas caries-free individuals have a more diverse flora with no subgroup predominance. Abnormalities in quantity or composition of the fecal microbiota have been linked to diseases such as ulcerative colitis [41], Crohn's disease[42,43], and celiac disease [44,45].

The association between atopic eczema and subsequent development of asthma in children is well recognized [46]. A recent study by Burgess et al showed that a history of childhood eczema is also associated with new-onset of asthma later in life and with asthma persisting into middle age [47]. Being able to identify infants at risk and prevent the development of eczema would not only lessen the burden of the disease itself, but could also help identify children at risk for and perhaps help prevent asthma in those infants later in childhood and into adulthood.

Non-culture dependent methods such as DGGE have better sensitivity to detect certain bacterial species than stool cultures, which may provide incomplete data because of inability to detect non-culturable bacteria. These new methods provide a more accurate view of the bacterial community and allow for the study of the whole system, rather than focusing on specific species. Although it would have been ideal to perform full genotyping and sequencing of bacterial DNA, we were limited by our sample size. Similarly, potential differences in the relation between microbial diversity and eczema by mode of delivery must be interpreted with caution because of the small sample size of our study. History of antibiotic usage by the infants was not associated with the diagnosis of eczema; although information was missing on 2 subjects (1 control and 1 case), sensitivity analysis for the missing data showed no change in the results.

## Conclusion

We found a significant inverse association between fecal bacterial diversity and eczema in early life. In addition, we found a significant increment in fecal bacterial diversity from ages 1 to 4 months in healthy children, but not in children who developed eczema by age 6 months. Further research is needed to investigate these findings, including larger sample sizes to elucidate the effect of time and other factors known to be associated with bacterial diversity and/or atopy, DGGE standardization and genotyping to identify groups of bacteria and patterns associated with the development of eczema, and long-term follow-up to provide information regarding development of other atopic diseases such as asthma.

## Competing interests

Please see additional file 1 which contains the disclosure for Dr. Scott T. Weiss. All other authors declare that they have no competing interests.

## Authors' contributions

EF participated in the data analysis and interpretation, and the preparation of the manuscript. JCC, AAL, DRG, and STW participated in the study design and data interpretation. JCC also participated in the manuscript preparation. DL participated in the coordination of the study and the collection of data. ABO, MLD and AMD processed the samples and performed the PCR and DGGE. JMcC and LMR participated in the data analysis. All authors read and approved the final manuscript.

## Supplementary Material

Additional file 1 Competing interests disclosure. Disclosure of competing interests for Dr. Scott T. Weiss Click here for file
