# Supplementary material for: Diversity of the gut microbiota and eczema in early life
Source: Clin Mol Allergy. 2008 Sep 22;6:11. doi: 10.1186/1476-7961-6-11 (PMC2562383; doi:10.1186/1476-7961-6-11)
Supplement: Additional file 1 — Competing interests disclosure. Disclosure of competing interests for Dr. Scott T. Weiss [file 1476-7961-6-11-S1.doc]

# Financial Disclosure Statement for Dr. Scott T. Weiss

February 2008

I certify that I have no affiliation with, nor have I any financial involvement in, any organization or entity resulting in a direct financial interest to me (e.g., employment, consultancy, stock ownership, honoraria, equity, etc.) concerning the manuscript or grant application discussed on the attached document(s), except as disclosed below.

Any financial support concerning the attached document(s) is acknowledged where appropriate (on manuscripts, grant applications, and other submitted documents).

Below I have indicated my commitment to various organizations with which I have had or currently have an affiliation that involves significant financial interest (in the form of grants, consultancies, speakers’ bureau, etc.):

| Organization Name | **Role(s)** | **Date(s)** |
| --- | --- | --- |
| Glaxo-Wellcome | Grant recipient and consultant | 2000 – 2005 |
| Roche Pharmaceuticals | Consultant | 2000-2002 |
| AstraZeneca | Grant recipient | 1997 – 2003 |
| Millennium Pharmaceuticals | Consultant | 1996 – 2001 |
| Genentech | Consultant/Chair, TENOR Board | 2001 – present |
| Pfizer | Grant recipient | 2000 – 2003 |
| Schering-Plough | Consultant | 1999 – 2000 |
| Variagenics | Consultant | 2002 |
| Genome Therapeutics | Consultant | 2003 |
| Merck Frost | Consultant | 2002 |

Signed:


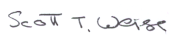
 02/07/2008

Scott T. Weiss, M.D., M.S. Date

Principal Investigator/Author/Co-Author

Professor of Medicine, Harvard Medical School

Director, Division of Respiratory, Environmental

and Genetic Epidemiology

Associate Director, Harvard-Partners Center

for Genetics and Genomics

Channing Laboratory

Brigham and Women’s Hospital
